# Editorial: New trends in green chemistry: sustainable and alternative strategies for the extraction of high-value compounds from agri-food matrices and residues

**DOI:** 10.3389/fchem.2026.1861325

**Published:** 2026-04-30

**Authors:** Francesca Annunziata, Gigliola Borgonovo, Patricia Rijo, Andrea Pinto

**Affiliations:** 1 Department of Food, Environmental and Nutritional Sciences (DeFENS), University of Milan, Milan, Italy; 2 CBIOS Lusófona’s Research Center for Biosciences and Health Technologies, Campo Grande, Lisbon, Portugal; 3 Faculty of Pharmacy, Research Institute for Medicines (iMed.ULisboa), Universidade de Lisboa, Lisbon, Portugal; 4 Centro de Química Estrutural, Institute of Molecular Sciences, Universidade de Lisboa, Lisboa, Portugal; 5 Faculty of Biology and Environmental Protection, University of Lodz, Lodz, Poland

**Keywords:** agro-food by-products, alternative strategies, green chemistry, high-value compounds, novel extraction methods

Every year millions of tons of waste are accumulated along the food production chain: plant-based residues like peels, leaves seeds, pomace, husks, pulps, are usually underused or discarded contributing to waste generation and environmental pollution (Gonçalves and Maximo). Beneath the surface, however, lies a rich molecular landscape: agri-food matrices and residues are naturally rich in valuable bioactive and relevant compounds. Phenolics, carotenoids, anthocyanins, vitamins, fibers, essential oils and a plethora of other phytochemicals have been extensively studied for their remarkable diversity of biological activities (‘Aqilah et al.).

This Research Topic, hosted by *Frontiers in Chemistry* within the Green and Sustainable Chemistry section, highlights recent advances in environmentally friendly and innovative strategies for the extraction and valorization of high-value compounds from agri-food matrices and residues. In response to the growing demand for sustainable solutions and circular economy approaches, this Research Topic brings together research focused on reducing environmental impact while enhancing the efficiency and applicability of extraction technologies.

Within the framework of green chemistry, the contributions in this Research Topic address critical challenges related to agri-food waste management and demonstrate how such residues can be revalorized into valuable bioactive compounds. The 5 articles included, comprising both original research and review work, provide a comprehensive perspective on emerging extraction methodologies, sustainable solvents, and innovative processing strategies.

Njewa et al., provide a thorough review of the extraction, isolation, and characterisation of bioactive compounds derived from agri-food waste. Their work highlights the technological and industrial relevance of these compounds, emphasizing the need for greener, scalable extraction processes that align with sustainability goals.

Tumminelli et al. investigate the valorisation of vineyard pruning-wood waste, demonstrating its potential as a sustainable and renewable source of bioactive molecules such as resveratrol and ε-viniferin. Their findings reinforce the concept of waste-to-value approaches within viticulture and the broader agri-food sector.

Gómez Vargas et al. explore the extraction of pectin from *Malus domestica* agro-industrial residues using green solvents such as citric acid and natural deep eutectic solvents (NADES). Their study shows that these alternatives can effectively replace conventional solvents while preserving the functional and physicochemical properties of the extracted biopolymer.

Yang et al. focus on corn processing by-products, revealing their hypoglycaemic and antioxidant properties. This work highlights the potential application of such residues in nutraceutical and functional food development, supporting their integration into value-added product chains.

El Jabali et al. examine the impact of high-intensity ultrasound combined with calcium chelating agents on casein micelles. Their research demonstrates how innovative and sustainable processing techniques can modulate protein structure and functionality, opening new avenues for food system design.

Overall, the studies presented in this Research Topic demonstrate the transformative potential of green chemistry in redefining the use of agri-food residues. By leveraging sustainable extraction technologies and alternative solvents, these works contribute to reducing waste and promoting a circular bioeconomy. Together, they illustrate the multifaceted potential of green extraction technologies and offer a roadmap for future research.

In conclusion, this Research Topic underscores the importance of sustainable and alternative extraction strategies in unlocking the value of agri-food by-products. The findings presented not only advance scientific knowledge but also provide practical insights for industrial implementation. As sustainability continues to shape the direction of scientific and technological development, the approaches highlighted here will be instrumental in guiding future innovations in green chemistry and resource management.
